# Leukotrienes inhibit early stages of HIV-1 infection in monocyte-derived microglia-like cells

**DOI:** 10.1186/1742-2094-9-55

**Published:** 2012-03-16

**Authors:** Jonathan Bertin, Corinne Barat, Dave Bélanger, Michel J Tremblay

**Affiliations:** 1 Centre de Recherche en Infectiologie, RC709, Centre Hospitalier Universitaire de Québec-CHUL, 2705 Boul. Laurier, Québec, QC G1V 4G2, Canada

**Keywords:** Central nervous system, Microglia, HIV-1, Leukotrienes

## Abstract

**Background:**

Microglia are one of the main cell types to be productively infected by HIV-1 in the central nervous system (CNS). Leukotriene B_4 _(LTB_4_) and cysteinyl-leukotrienes such as LTC_4 _are some of the proinflammatory molecules produced in infected individuals that contribute to neuroinflammation. We therefore sought to investigate the role of leukotrienes (LTs) in HIV-1 infection of microglial cells.

**Methods:**

To evaluate the role of LTs on HIV-1 infection in the CNS, monocyte-derived microglial-like cells (MDMis) were utilized in this study. Leukotriene-treated MDMis were infected with either fully replicative brain-derived HIV-1 isolates (YU2) or R5-tropic luciferase-encoding particles in order to assess viral production and expression. The efficacy of various steps of the replication cycle was evaluated by means of p24 quantification by ELISA, luciferase activity determination and quantitative real-time polymerase chain reaction (RT-PCR).

**Results:**

We report in this study that virus replication is reduced upon treatment of MDMis with LTB_4 _and LTC_4_. Additional experiments indicate that these proinflammatory molecules alter the pH-independent entry and early post-fusion events of the viral life cycle. Indeed, LT treatment induced a diminution in integrated proviral DNA while reverse-transcribed viral products remained unaffected. Furthermore, decreased C-C chemokine receptor type 5 (CCR5) surface expression was observed in LT-treated MDMis. Finally, the effect of LTs on HIV-1 infection in MDMis appears to be mediated partly via a signal transduction pathway involving protein kinase C.

**Conclusions:**

These data show for the first time that LTs influence microglial cell infection by HIV-1, and may be a factor in the control of viral load in the CNS.

## Background

Infection of the central nervous system (CNS) by human immunodeficiency virus type 1 (HIV-1) causes neurotoxicity and inflammatory disorders such as encephalitis and associated neurocognitive deficits [1]. Since the introduction of highly active antiretroviral therapy (HAART) as the standard of care for seropositive patients, the incidence of severe neurological complications caused by HIV-1 such as dementia has greatly diminished, but a more subtle form of CNS dysfunction known as minor cognitive and motor disorder has emerged [2]. Although a discordance between cerebrospinal fluid (CSF) and plasma viral load is often observed, sustained virus replication in the CNS despite suppressive antiretroviral therapy correlates with acute or subacute neurological alterations [3-5]. Moreover, the viral strains present in the CNS may, over time, genetically differ from virus isolates circulating in peripheral blood [6]. Unsuppressed virus replication in the brain favors the emergence of drug-resistant viruses and the appearance of viral variants with reduced dependence for CD4, the primary cellular receptor for HIV-1 [7]. Nevertheless, some studies have shown that the level of CNS penetration in patients receiving HAART does not significantly influence clinical efficacy [1,8], although this issue remains controversial [9].

Entry of HIV-1 into the CNS occurs very early after primary infection [10]. The Trojan horse model proposes that HIV-1 can cross the blood-brain barrier (BBB) through monocytes carrying virions and then be liberated into the CNS [11-13]. Also, it has been postulated that elevated concentrations of the bacterial endotoxin lipopolysaccharide (LPS) in the plasma of HIV-1-infected patients, in combination with several cytokines, viral proteins and eicosanoid lipid mediators, increase permeability of the BBB, thus facilitating invasion of HIV-1 into the CNS [11,14,15]. There, perivascular macrophages and microglia, along with astrocytes to some extent, are the cells permissive to HIV-1 infection; they also act as long-lived viral reservoirs despite antiviral therapies [6,16-20].

Leukotrienes (LTs), which are classified as eicosanoids, are among the first soluble factors produced by the innate immune system when challenged by an invading pathogen. These potent proinflammatory lipid mediators display strong vasoconstrictive, chemotactic and proliferative properties. They are associated with different inflammatory diseases, such as asthma, rhinitis and atherosclerosis. In stimulated cells, the enzyme 5-lipoxygenase (5-LO) initiates synthesis of LTs from arachidonic acid. A short-lived LT precursor, LTA_4_, is converted into either leukotriene B_4 _(LTB_4_) or cysteinyl-LTs (cysLTs), the latter includes LTC_4_, LTD_4 _and LTE_4_. CysLTs engage CysLT-1, CysLT-2 and GPR17 receptor subtypes, whereas LTB_4 _exerts its biological action by means of cell surface receptors denoted as BLT1 and BLT2, all of which belong to the family of G-protein-coupled receptors [21,22]. BLT1 is expressed primarily in leukocytes while BLT2 is expressed more ubiquitously [23,24]. It should be stated that both CysLT-1 and CysLT-2 are expressed in many cell types, including cells of the monocyte/macrophage lineage [25]. Engagement of LT receptors on microglia is associated with release of the multifunctional nucleotide adenosine-5'-triphosphate (ATP) and of cysLTs [26]. Furthermore, the increase of LT synthesis in microglial cells following LT-receptor activation requires the intracellular second messenger p38 MAP kinase (MAPK) [27].

Several studies have reported that LTs, along with their cognate receptors, are expressed in the brain [22,26,28-35]. Moreover, *in vitro *cocultures of HIV-1-infected monocytes and astroglia secrete high levels of eicosanoids such as LTB_4 _and LTD_4_, which largely contribute to neuroinflammation and neuronotoxicity [36]. *In vivo*, it has been reported that elevated amounts of LTB_4 _can be found in the CSF of patients with AIDS [37]. Moreover, recent studies have shown that increased levels of 5-LO and high concentrations of eicosanoids, including LTB_4_, are found in the brain of HIV-1 transgenic rats [38,39]. Moreover, it has been demonstrated that activated microglia can produce several proinflammatory cytokines, chemokines and certain eicosanoid metabolites [29,40-42]. In turn, chemotactic molecules secreted in neuropathologic conditions, such as LTs, contribute to the recruitment of potentially infected leukocytes (including monocytes and lymphocytes) into the CNS [43]. However, LTs have also been reported to indirectly exert anti-HIV-1 properties. For example, LTB_4 _induces the release by neutrophils of antimicrobial proteins, including α-defensins, that inhibit HIV-1 infectivity [44,45].

We therefore tested whether LTs can modulate virus infection in human microglial cells, which are known to play a pivotal role in the neuropathogenesis of HIV-1. Studies were performed using a previously described experimental cell system based on primary human monocyte-derived microglial-like cells (MDMis). We report herein that LTB_4 _and LTC_4 _negatively modulate some early events in HIV-1 infection in MDMis and that this effect relies at least partly on protein kinase C (PKC).

## Methods

### Reagents

LTB_4_, LTC_4 _and their respective less active metabolites (that is, *N*-acetyl LTE_4 _and 20-carboxy-LTB_4_) were all purchased from Cayman Chemical (Ann Arbor, MI, USA). Granulocyte macrophage colony-stimulating factor (GM-CSF) was a generous gift from Cangene (Winnipeg, Manitoba, Canada), whereas macrophage colony-stimulating factor (M-CSF) was purchased from GenScript Corporation (Piscataway, NJ, USA). The luciferase activity assay was carried out using the D-luciferin reagent purchased from Thermo Fisher Scientific (Rockford, IL, USA). Slot blots to quantify substance P in cell-free supernatants were revealed with the Western lightning plus ECL purchased from PerkinElmer (Waltham, MA, USA). The PKC inhibitor bisindolylmaleimide IX (also called Ro318220) was obtained from EMD Chemicals (Gibbstown, NJ, USA). The two LT receptor antagonists LY293111 and Bay-u9773 were purchased from Cayman Chemical.

### Antibodies

The anti-HIV-1 p24 hybridomas 31-90-25 and 183-H12-5C were obtained from the American Type Culture Collection (ATCC) (Manassas, VA, USA) and the NIH AIDS Repository Reagent Program (Bethesda, MD, USA), respectively. Antibodies were purified by using MAbTrap protein affinity columns according to the manufacturer's instructions (Amersham Pharmacia Biotech AB, Uppsala, Sweden). Differentiation of primary human monocytes into MDMis was assessed using an antibody specific for the ionized calcium binding adaptor protein 1 (IBA1) (Wako Chemical, Richmond, VA, USA) followed with a goat anti-rabbit antibody labeled with the Alexa Fluor^® ^555 dye (Molecular Probes, Eugene, OR, USA). Detection of substance P was assessed using the rabbit polyclonal anti-substance P antibody (Abbiotech, San Diego, CA, USA) and the horseradish peroxidase (HRP)-conjugated goat anti-rabbit antibody (Jackson Immunoresearch, West Grove, PA, USA). LT receptor expression was determined by flow cytometry using either a rabbit anti-cysLT2 antibody (Cayman Chemicals) followed by a goat anti-rabbit antibody conjugated with Alexa Fluor^® ^488 (Molecular Probes) or a biotinylated anti-BLT1 antibody (Cayman Chemical) followed by a streptavidin-R-phycoerythrin conjugate (BD Bioscience, Mississauga, Canada). C-C chemokine receptor type 5 (CCR5) expression on MDMis was assessed using the R-phycoerythrin-conjugated anti-CCR5 antibody (clone 2D7) purchased from BD Bioscience.

### Plasmids

The brain-derived full-length R5-tropic YU2 and X4-using NL4-3 infectious molecular clones of HIV-1 were obtained through the AIDS Research and Reference Reagent Program (pYU2 from Dr Beatrice Hahn and Dr George Shaw and pNL4-3 from Dr Malcolm Martin) [46,47]. The pNL4-3Bal*env *is a full-length infectious molecular clone of HIV-1 (kindly provided by R Pomerantz, Thomas Jefferson University, Philadelphia, PA, USA) [48]. This virus is an R5-using viral strain where the NL4-3 *env *gene has been replaced with the *env *gene from the R5-tropic Bal variant. As for the NL4-3*Luc*^+^*Env*^-^*R*^+ ^vector, kindly provided by Dr NR Landau (NYU School of Medicine, New York, NY, USA), it produces envelope-deficient HIV-1 particles coding for the *luciferase *reporter gene. Single-cycle reporter viruses were pseudotyped with JR-FL envelope or vesicular stomatitis virus envelope glycoprotein G (VSV-G) leading to NL4-3*Luc*^+^*Env*^-^*R*^+^/JR-FL and NL4-3*Luc*^+^*Env*^-^*R*^+^/VSV-G pseudotypes, respectively. The R5-tropic envelope-encoding JR-FL*env *vector was kindly supplied by Dr NR Landau, whereas pHCMV-G, which was obtained from the AIDS Repository Reagent Program, codes for the broad host-range VSV-G under the control of the human cytomegalovirus promoter.

### Cell culture

In order to obtain astrocyte-conditioned medium (ACM), normal human astrocytes were cultured in astrocyte basal medium supplemented with astrocyte growth medium. Primary human astrocytes and media were purchased from Lonza (Walkersville, MD, USA). At 80% cellular confluence, the medium was harvested, filtered (0.22 μm) and aliquoted. Aliquots of all passages were kept at -80°C up to the tenth passage. This ACM was then tested to determine the optimal percentage for differentiating primary human monocytes into MDMis. Briefly, peripheral blood mononuclear cells (PBMCs) from healthy donors were isolated by Ficoll-Hypaque gradient (Wisent Inc., St-Bruno, Qc, Canada). Next, cells were plated at a final concentration of 1 × 10^7 ^cells/ml in 75 cm^2 ^flasks (BD Bioscience, Mississauga, ON, Canada) for 2 h in order to separate by adherence monocytes from the other non-adherent cells. After washing with endotoxin-free phosphate-buffered saline (PBS) (Sigma-Aldrich, Oakville, ON, Canada), monocytes were cultured in complete RPMI-1640 medium (that is, RPMI supplemented with 10% heat-inactivated fetal bovine serum (FBS), 100 U/ml penicillin G and 100 μg/ml streptomycin) in the presence of M-CSF (10 ng/ml) and GM-CSF (1 ng/ml) for 2 days. Cells were then recovered with a soft cell scraper and plated in 24-well plates (BD Bioscience, Mississauga, ON, Canada) at a final concentration of 1 × 10^5 ^cells per well in complete RPMI-1640 medium supplemented with M-CSF (10 ng/ml), GM-CSF (1 ng/ml) and with (to obtain MDMis) or without (to be used as control cells) an optimized percentage of ACM (ranging from 25% to 35%). To confirm that ACM-treated monocytes display a cell phenotype resembling microglial-like cells, after 14 days of exposure to ACM the morphology of non-fixed cells was examined by phase-contrast microscopy (Nikon Eclipse TE300). After differentiation, MDMis were cultured in RPMI supplemented with 10% heat-inactivated FBS, 100 U/ml penicillin G and 100 μg/ml streptomycin (Invitrogen, Burlington, ON, Canada).

Human embryonic kidney 293T (ATCC) and TZM-bl cells (NIH AIDS Research and Reference Reagent Program) were cultured in complete Dulbecco's modified Eagle's medium (DMEM) (that is, DMEM supplemented with 10% heat-inactivated FBS, 2 mM L-glutamine, 100 U/ml penicillin G and 100 μg/ml streptomycin).

### Immunofluorescence microscopy for microglia characterization

Cells were seeded onto glass coverslips (12 mm round, thickness #1, Fisher Scientific, Nepean, ON, Canada) set in 24-well plates at 1 × 10^5 ^cells per well and treated with ACM for 14 days 2 days after peripheral blood monocytes were isolated and incubated in the presence of M-CSF and GM-CSF. Once monocytes were derived into MDMis, cells were first fixed with 4% (w/v) room temperature paraformaldehyde-PBS for 20 minutes and next treated with a blocking solution (PBS supplemented with 1% bovine serum albumin (BSA), 0.2% Triton, 30% normal goat serum and 15% human serum) for 15 minutes followed by an incubation with a rabbit anti-IBA1 antibody (diluted 1:500) in PBS containing 1% BSA at room temperature for 1 h. After washing, cells were incubated with goat anti-rabbit antibody conjugated with Alexa-555 (dilution 1:500) at room temperature for 30 minutes. After washing, coverslips were mounted on microscope slides using Fluoromount-G from Southern Biotech (Birmingham, Alabama, USA) and dried overnight in the dark. Cells were observed using a Nikon Eclipse TE300 microscope.

### Virus production

Viruses were produced by the calcium phosphate coprecipitation method in 293T cells as described previously [49]. In brief, 293T cells were transiently transfected with pYU2 to produce fully infectious R5-tropic HIV-1 particles. In addition, 293T cells were cotransfected with pNL4-3*Luc*^+^*Env*^-^*R*^+ ^and either pJR-FL*env *or pHCMV-G to obtain pseudotyped HIV-1-based reporter viruses. Virus preparations were normalized for virion content by using an in-house enzymatic assay specific for the major viral p24 protein. In this test, hybridomas 183-H12-5C and 31-90-25 are used in combination to quantify p24 levels [50]. In addition, the infectivity of our virus stocks was assessed using TZM-bl indicator cells. This cell line is a genetically modified HeLa-derived cell line expressing large amounts of cell surface CD4, CCR5 and C-X-C chemokine receptor type 4 (CXCR4) [51]. These cells carry separate integrated copies of the luciferase and β-galactosidase genes under the control of the HIV-1 promoter and are highly susceptible to infection with different HIV-1 variants (both R5 and X4 tropic). Virus preparations that did not allow sufficient reporter gene activity were discarded.

### HIV-1 infection

After cell differentiation, MDMis were washed extensively with PBS, plated at 1 × 10^5 ^cells per well were and incubated in a final volume of 400 μl of RPMI supplemented with 10% heat-inactivated FBS, 100 U/ml penicillin G and 100 μg/ml streptomycin. Cells were treated with LTs either before (45 minutes) or after infection (2, 24, or 48 h) with luciferase-encoding pseudotyped viruses. As for infections using a fully replicative virus (that is, YU2), cells were treated with LTs both before and after infection where a fraction of the medium was removed (50 μl) every 3 days and for a period lasting 9 days and kept frozen at -20°C. Virus infection was performed with a fixed amount of virus (that is, 10 ng of p24 per 1 × 10^5 ^cells) for 2 h and cells were washed to remove excess virus. Virus production was estimated by measuring p24 levels in cell-free culture supernatants by ELISA. Luciferase activity was measured 7 days after infection using an MLX microtiter luminometer (Dynex Technologies, Chantilly, VA, USA) and is expressed as relative luciferase units (RLU). In some experiments, cell-free supernatants from MDMis either left untreated or treated with LTs and next infected with HIV-1 for 9 days were collected and virus infectivity was defined upon incubation with TZM-bl indicator cells.

### Flow cytometry analysis

Before staining, cells were incubated for 15 minutes at 4°C in PBS containing 0.1% sodium azide, 10% heat-inactivated human fibrin-depleted plasma, 10% normal goat serum (Jackson ImmunoResearch Laboratories, West Grove, PA, USA) and 10% FBS to block non-specific binding sites and washed once with PBS containing 0.1% sodium azide and supplemented with 5% FBS. To monitor cell surface expression of CCR5, BLT1, BLT2, cysLT1 and cysLT2, MDMi samples, treated or not with LTs as indicated, were incubated with specific antibodies or with an appropriate isotype-matched irrelevant control antibody (for non-specific staining) for 30 minutes at 4°C, or for 20 minutes at room temperature exceptionally for CCR5. Cells were then washed with PBS supplemented with 0.1% sodium azide and 5% FBS and fixed in a 2% paraformaldehyde solution before analysis by flow cytometry (Epics ELITE ESP; Coulter Electronics).

### Substance P detection

Cell-free supernatants were obtained from monocytes cultured with or without ACM and blotted onto a 0.2 μm polyvinylidene fluoride (PVDF) membrane (Millipore, Billerica, MA, USA) using a vacuum slot blotter. The membrane was then blocked by incubation in PBS containing 5% dried milk and 0.2% Tween-20 for 2 h and then probed with polyclonal rabbit antibody to substance P (1:500) at 4°C overnight. Membranes were then washed four times for 15 minutes in PBS containing 0.2% Tween-20 and incubated for 1 h with HRP-conjugated goat-anti-rabbit IgG (1:20,000). After 2 h of washing in PBS containing 0.2% Tween-20, the reaction was visualized with the enhanced chemiluminescence system (ECL plus) as recommended by the manufacturer. Signal intensity on the film was quantified with densitometric analysis software (ImageJ).

### Real-time polymerase chain reaction (PCR)

For detection and quantification of reverse transcripts and integrated viral DNA copies, MDMis (1 × 10^6^) were pulsed for 2 h with a virus preparation (that is, YU2) previously treated with 20 U/ml grade II DNase-1 (Roche Applied Science, Qc, Canada). Cells were then either left untreated or treated for 8 h (transcripts) or 24 h (proviral DNA) with LTs (10 ng/ml). Genomic DNA was extracted using the DNeasy Blood and Tissue Kit from Qiagen (Toronto, ON, Canada). Quantification of HIV-1 reverse transcripts was achieved by real-time PCR in a 25 μl reaction containing 25 ng of DNA, TaqMan Universal PCR Master Mix (Applied Biosystems, Foster City, CA, USA), 1 μM each of HIV-1-specific sense M667 and antisense M661 primers and 0.3 μM of the TaqMan probe HIV-5'-carboxyfluorescein (Biosearch Technologies, Novato, CA, USA) [52]. For monitoring integrated viral DNA copies, DNA was subjected to a combined *Alu*-HIV-1 PCR and real-time PCR as described previously [53]. Briefly, genomic DNA (100 ng) was first amplified with an *Alu*-sequence-specific sense primer and HIV-1-specific antisense primer (that is, M661) [54]. Next, 5 μl of 20-fold diluted PCR products were subjected to a real-time PCR assay in a 25 μl reaction containing TaqMan Universal PCR Master Mix, 2 μM of the HIV-1-specific sense primer M667, 2 μM of the HIV-1-specific antisense primer AA55 and 0.3 μM of the TaqMan probe HIV-5'-carboxyfluorescein (Biosearch Technologies) [53]. We used a standard curve made of known amounts of input viral DNA (that is, pNL4-3 ranging from 469 to 30,000 copies). All HIV-1 standards contained 1 ng of DNA from uninfected cells as carrier. To quantify 2LTR circles, 125 ng of DNA per 5 μl was subjected to a real-time PCR reaction containing TaqMan Universal PCR Mix, 2 μM of the HIV-1 2LTR-specific primers (sense: CCCTCAGACCCTTTTAGTCAGTG; antisense: TGGTGTGTAGTT CTGCCAATCA) and 0.3 μM of the TaqMan probe 2LTR-FAM (GGA TCT ACC ACA CAC AAG GCT TCC) [53]. To ensure quantification precision, all HIV-1 real-time PCR amplified samples were normalized using the β-globin housekeeping gene. From every diluted DNA samples, β-globin was quantified using 1 μM of sense (TGGTCTATTTTCCCACCCT) and antisense (TGGCAAAGGTGCCCTTGA) specific primers and 0.3 μM of the TaqMan probe 5'-β-globin-CAL Fluor 560 (TCTGTCCACTCCTGATGCTG-BHQ-1 3') (Biosearch Technologies). Equal parts of every sample were pooled and successive twofold dilutions were used as a relative standard curve for β-globin quantification. The cycling conditions used for the Applied Biosystems 7500 sequence detection system included a hot start (50°C for 2 minutes and 95°C for 10 minutes), followed by 40 cycles of denaturation (95°C for 1 minute) and extension (63°C for 1 minute) with end point acquisition.

### Statistical analysis

All experiments were repeated at least three times and each figure combines the results obtained with all the different donors unless otherwise specified. The statistical significance of the difference between groups was determined using the Student t test. Calculations were made with Prism version 3.03 (GraphPad Software, Inc. La Jolla, CA, USA). The *P *values < 0.05 were considered statistically significant.

## Results

Because of the limited access to primary microglial cells isolated from human brain specimens, which renders experimental approaches that require large quantities of cells difficult to achieve, we turned to a previously described experimental model system to study the possible modulatory role of LTs in HIV-1 infection in microglia. This unique cellular model is capable of supporting productive infection with HIV-1 [55]. Furthermore, this *in vitro *model mimics infiltrating microglia [56,57], one of the cell types mostly responsible for disseminating HIV-1 in the CNS.

### Validation of the cellular model

**Figure 1:**
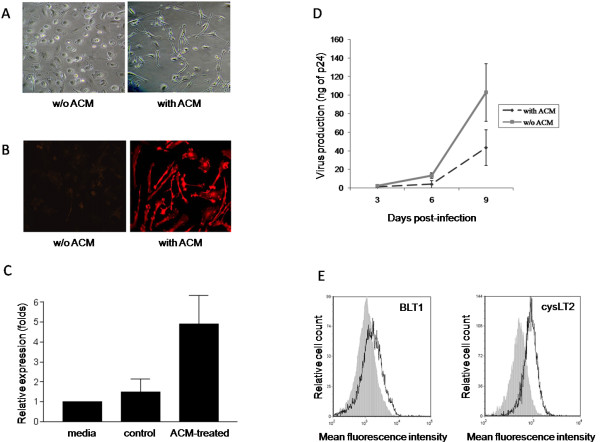
**Characterization of microglial-like cells**. Monocytes were isolated from peripheral blood mononuclear cells (PBMCs) of healthy donors, and then cultured either in absence or presence of astrocyte-conditioned medium (ACM). **(A) **Cell morphology was observed by phase-contrast microscopy. Monocytes cultured in the absence of ACM were used as control cells. The results shown are representative of at least three different donors. **(B) **ACM-differentiated cells were permeabilized and stained with anti-ionized calcium binding adaptor protein-1 (IBA1) antibodies. IBA1 expression was observed by epifluorescence microscopy. The results shown are representative of at least three distinct donors. **(C) **Substance P secretion by monocyte-derived microglial-like cells (MDMis) and non-ACM-treated control cells was evaluated by slot-blot. Results from two independent donors (means ± SEM) are presented. **(D) **ACM-treated and non-treated monocytes were infected with YU2. Virus replication was assessed by monitoring the p24 content at days 3, 6 and 9 post infection. The means ± SEM are calculated from three independent experiments with triplicate samples. **(E) **The surface expression of BLT1 and cysLT2 receptors on ACM-treated monocytes was determined by flow cytometry. The results shown represent a single donor out of a total of four.

Microglial-like cells are characterized by a typical quiescent microglia morphology consisting in long branching processes and a small cellular body. These cells also express IBA1, which is an intracellular molecule marker known to be abundantly expressed in microglia [58]. To validate our cellular model, we first examined the morphology of the cells, as well as their IBA1 expression level. As expected, ACM-treated monocytes display typical microglia morphology following differentiation (Figure 1A) and express much higher levels of IBA1 than non-ACM-treated cells (used as control cells) (Figure 1B). Substance P, the most abundant neurokinin in the CNS, is secreted in significantly higher amounts by microglia than monocyte-derived macrophages [59]. Indeed, our MDMi preparations were found to produce considerably more substance P than non-ACM treated monocytes (Figure 1C). Furthermore, MDMis were found to be permissive to productive infection with the brain-derived R5-using HIV-1 isolate YU2, but produced less progeny virus than non-ACM-treated monocytes (Figure 1D), which is in line with previous published observations [55]. In addition, MDMis were found to be refractory to infection with a prototypic X4-using HIV-1 variant (that is, NL4-3) (data not shown). Altogether, these data show that our cellular model displays the hallmarks of microglial-like cells.

We also analyzed the surface expression of LTB_4 _(that is, BLT1 and BLT2) and cysLT receptors (that is, cysLT1 and cysLT2) on MDMis. In our study, neither BLT2 nor cysLT1 were detected on MDMis by flow cytometry (data not shown). However, BLT1 and CysLT2 were found to be expressed on these cells, albeit at a moderate level, with mean fluorescence intensities of 2,200 and 993, respectively (Figure 1E).

### LTs decrease HIV-1 replication in MDMis

**Figure 2:**
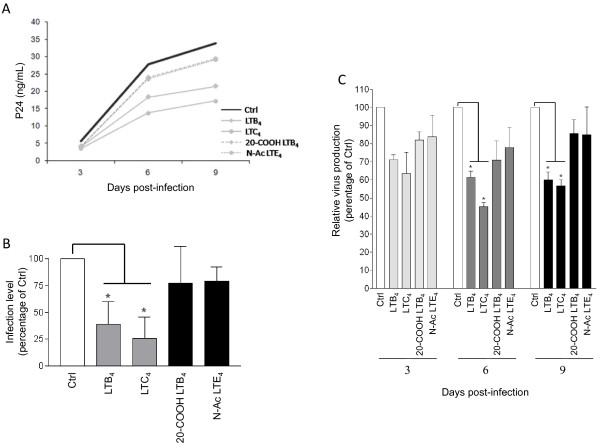
**Leukotrienes (LTs) reduce susceptibility of monocyte-derived microglial-like cells (MDMis) to HIV-1 infection**. **(A) **MDMis were pretreated for 45 minutes with 100 ng/ml of the indicated LTs prior to infection with YU2. At 2 h after infection, cells were then thoroughly washed and incubated in fresh media containing the listed LTs. Additional LTs were also replenished at days 3 and 6 post infection. Viral production was monitored at 3, 6 and 9 days post infection by measuring the p24 content. The results shown are from one representative donor of a total of three. **(B) **The p24 values for all three donors tested are depicted as percentage of untreated control to compensate for donor-to-donor virus infection variability. **(C) **Production of infectious virus particles by LT-treated MDMis was quantified at 9 days post infection in cell-free supernatants using the TZM-bl indicator cell line. Results shown represent mean luciferase activity ± SD calculated from three independent experiments with triplicate samples and are expressed as the relative percentage of infectious virus production with respect to non-treated cells (*P < 0.05).

We next evaluated if LTs could modulate HIV-1 replication in MDMis. To do so, MDMis were initially treated, or not, with either LTB_4 _or LTC_4 _and then infected with the fully replicative R5-tropic strain YU2. Viral production was monitored for a period of 9 days. It is noteworthy that LTs were replenished during the experiment (that is, at days 3 and 6) because they are known to be very unstable. To make sure that the effect of LTs on HIV-1 infection was receptor specific, we used as controls the two less active metabolites 20-carboxy-LTB_4 _(called 20-COOH LTB_4_) and *N*-acetyl LTE_4 _(called *N*-Ac LTE_4_). For example, the biological activity of 20-carboxy-LTB_4 _is only 2.6% of that of LTB_4 _in causing human leukocyte degranulation [60], whereas *N*-Ac LTE_4 _is 100 times less potent than LTC_4 _as a vasoconstricting agent [61]. Treatment of MDMis with LTB_4 _and LTC_4 _causes a diminution in HIV-1 infection, as assessed by p24 values, to a greater extent than when using the less potent catabolites (Figure 2A, B). To validate whether the LT-mediated decrease in p24 levels correlates with a diminution of virus infectivity, supernatants were transferred onto TZM-bl indicator cells. The relative infectivity was assessed by comparing the luciferase activity generated by supernatants from YU2-infected (that is, 9 days post infection) MDMis either left untreated or treated with LTs or the less active analogs. Data from Figure 2C revealed that production of infectious virus is reduced in a more significant manner by LTs (that is, 61% reduction with LTB_4 _and 74% diminution with LTC_4_) compared to MDMis treated with their less active analogs (20-COOH LTB_4 _and *N*-Ac LTE_4_, respectively), which is in agreement with p24 values. The colorimetric MTS assay revealed no toxicity mediated by LTs in MDMis at the highest concentration tested (that is, 100 ng/ml) for a period of 9 days (data not shown).

### LTB_4 _and LTC_4 _inhibit early steps in HIV-1 replication in MDMis

**Figure 3:**
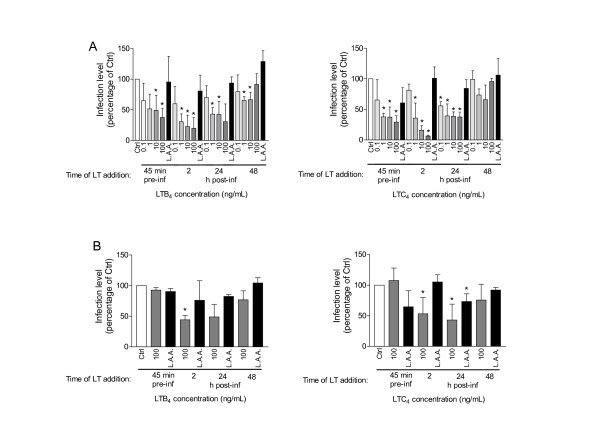
**Leukotrienes (LTs) exert an inhibitory effect on the early stages of HIV-1 infection in monocyte-derived microglial-like cells (MDMis)**. Cells were either left untreated, or treated with increasing concentrations of LTB_4 _(left panels) or LTC_4 _(right panels), either 45 minutes before virus exposure or 2 h to 48 h post infection, as indicated. Cells were first washed extensively with phosphate-buffered saline (PBS) and then infected at 45 minutes after addition of LTs. MDMis were washed a second time at 2 h after infection before adding LTs. No washes were carried out before adding LTs at 24 h and 48 h. Infection was carried out with a luciferase encoding reporter virus pseudotyped with JR-FL **(A) **or vesicular stomatitis virus envelope glycoprotein G (VSV-G) envelope **(B)**. The less active analogs (called LAA) 20-carboxy-LTB_4 _and *N*-acetyl LTE_4 _were used as negative controls for LTB_4 _and LTC_4_, respectively. The extent of virus infection was estimated by measuring virus-encoded luciferase activity at 7 days post infection. The results shown represent the means ± SD calculated from three, or four (bottom right panel), independent experiments with triplicate samples and are expressed as the percentage of luciferase activity with respect to non-treated cells (*P < 0.05).

We next sought to determine which step(s) of the HIV-1 life cycle LTs affected in MDMis. This goal was achieved by performing a timecourse experiment with luciferase-encoding single-cycle reporter viruses. Moreover, to define whether LTs can alter the process of virus entry, MDMis were inoculated with both R5 (that is, JR-FL envelope) and VSV-G pseudotyped reporter viruses. This is based on the idea that VSV-G pseudotypes enter cells by a pH-dependent endocytosis process and independently of the natural pH-independent pathway that relies on HIV-1 receptor and coreceptors. As shown in Figure 3, treatment of MDMis with LTs prior to virus infection reduced infection with JR-FL pseudotypes in a dose-dependent manner (A), whereas infection with reporter viruses pseudotyped with VSV-G was left unaffected (B), therefore suggesting that the initial steps in the replicative cycle of HIV-1 in MDMis are negatively affected by LTs. However, infection by both JR-FL and VSV-G pseudotypes was reduced when LTs were added to MDMis at 2 h or 24 h post infection (with the strongest effect seen at 2 h post infection), thus suggesting that a post-fusion step is also affected by LT treatment. The late infection stages (that is, after viral integration) are not touched by LTs since no significant modulatory effect was seen when cells were treated at a later time point (that is, 48 h post infection). Although a greater inhibition was observed when using a final concentration of 100 ng/ml of LTs, the more physiological concentration of 10 ng/ml was used in subsequent experiments.

**Figure 4:**
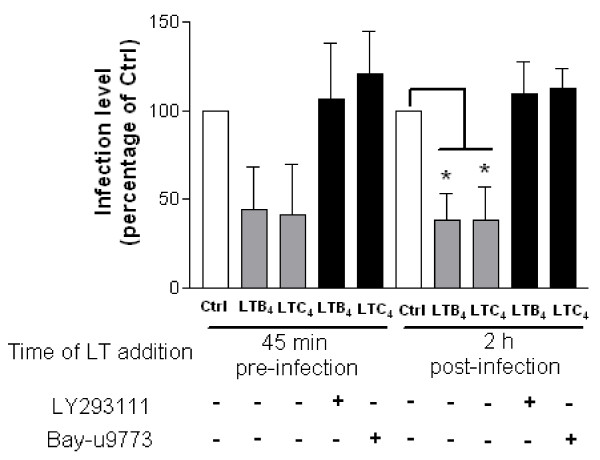
**Leukotriene (LT) receptor antagonists block the modulatory effect of LTB_4 _and LTC_4 _on HIV-1 replication in monocyte-derived microglial-like cells (MDMis)**. Cells were either left untreated (Ctrl) or treated with the listed LT receptor antagonists (that is, LY293111 or Bay-u9773) for 45 minutes before being thoroughly washed. MDMis were then either exposed to LTs 45 minutes before or 2 h following HIV-1 infection, after which cells were thoroughly washed. Virus infection was carried out using JR-FL pseudotyped viruses. The extent of virus infection was estimated by measuring virus-encoded luciferase activity at 7 days post infection. The results shown represent the means ± SD calculated from three independent experiments with triplicate samples and are expressed as the percentage of luciferase activity with respect to non-treated cells (*P < 0.05).

To further validate that LTB_4 _and LTC_4 _must engage their respective receptors in order to modulate the early steps of HIV-1 replication, MDMis were treated with potent LT receptor antagonists prior to LT treatment and infection with JR-FL pseudotypes. To this end, LY293111 was used to block BLT1 while Bay-u9773 was used to block cysLT1/2. Results show that the LTB_4_/LTC_4_-induced inhibition of viral production in MDMis is totally abolished by LY293111 or Bay-u9773, respectively (Figure 4).

### LTB_4 _and LTC_4 _downregulate surface expression of CCR5 on MDMis

**Figure 5:**
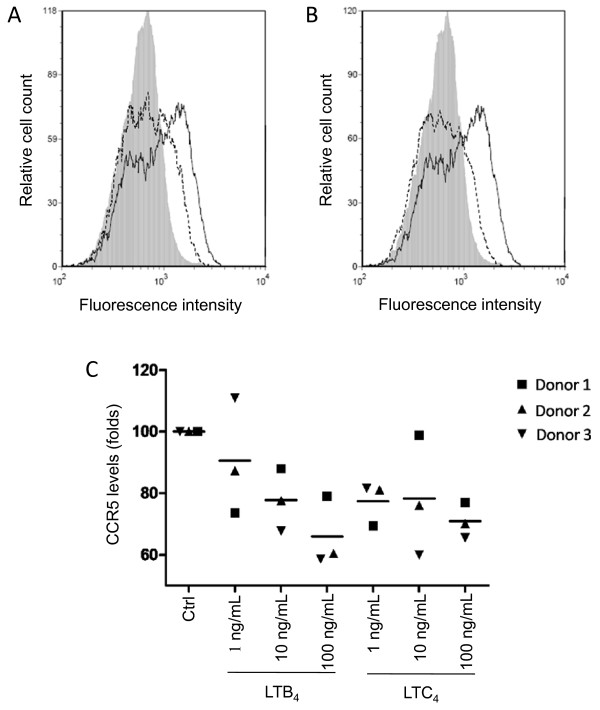
**Leukotriene (LT)B_4 _and LTC_4 _induce a downmodulation of C-C chemokine receptor type 5 (CCR5) surface expression on monocyte-derived microglial-like cells (MDMis)**. Cells were either left untreated (continuous lines) or treated with 10 ng/ml of **(A) **LTB_4 _or **(B) **LTC_4 _(dotted lines) for 24 h before labeling with a phycoerythrin (PE)-conjugated anti-CCR5 antibody or PE-conjugated irrelevant control antibody (gray areas). Expression levels of CCR5 were determined by flow cytometry. Results shown are from one donor representative of three. **(C) **MDMis were either left untreated or treated with increasing concentrations of LTs for 24 h before labeling with a PE-conjugated anti-CCR5 antibody. To compensate for donor-to-donor variations, the results shown represent the mean percentage of CCR5 positive cells ± SD calculated from three independent experiments and are expressed as percentages of control (Ctrl) (*P < 0.05).

Because pretreatment of MDMis with LTs affects infection by JR-FL but not VSV-G pseudotypes, we hypothesized that the pH-independent fusion step of the virus life cycle is likely inhibited by LTs. We investigated whether this could occur via a decrease in the expression of the HIV-1 coreceptor CCR5. Results obtained by flow cytometry show that incubation of MDMis in the presence of LTB_4 _or LTC_4 _for a 24-h period caused a diminution in the percentage of MDMis expressing CCR5 at the cell surface (Figure 5). Meanwhile, CCR5 expression remained unaffected after a 45-minute incubation period (data not shown), which points to an indirect effect on CCR5, rather than a direct receptor heterologous desensitization. These data suggest that LTs likely inhibit productive HIV-1 infection in MDMis partly via a modulation of CCR5 surface expression.

### LTs reduce virus integration

**Figure 6:**
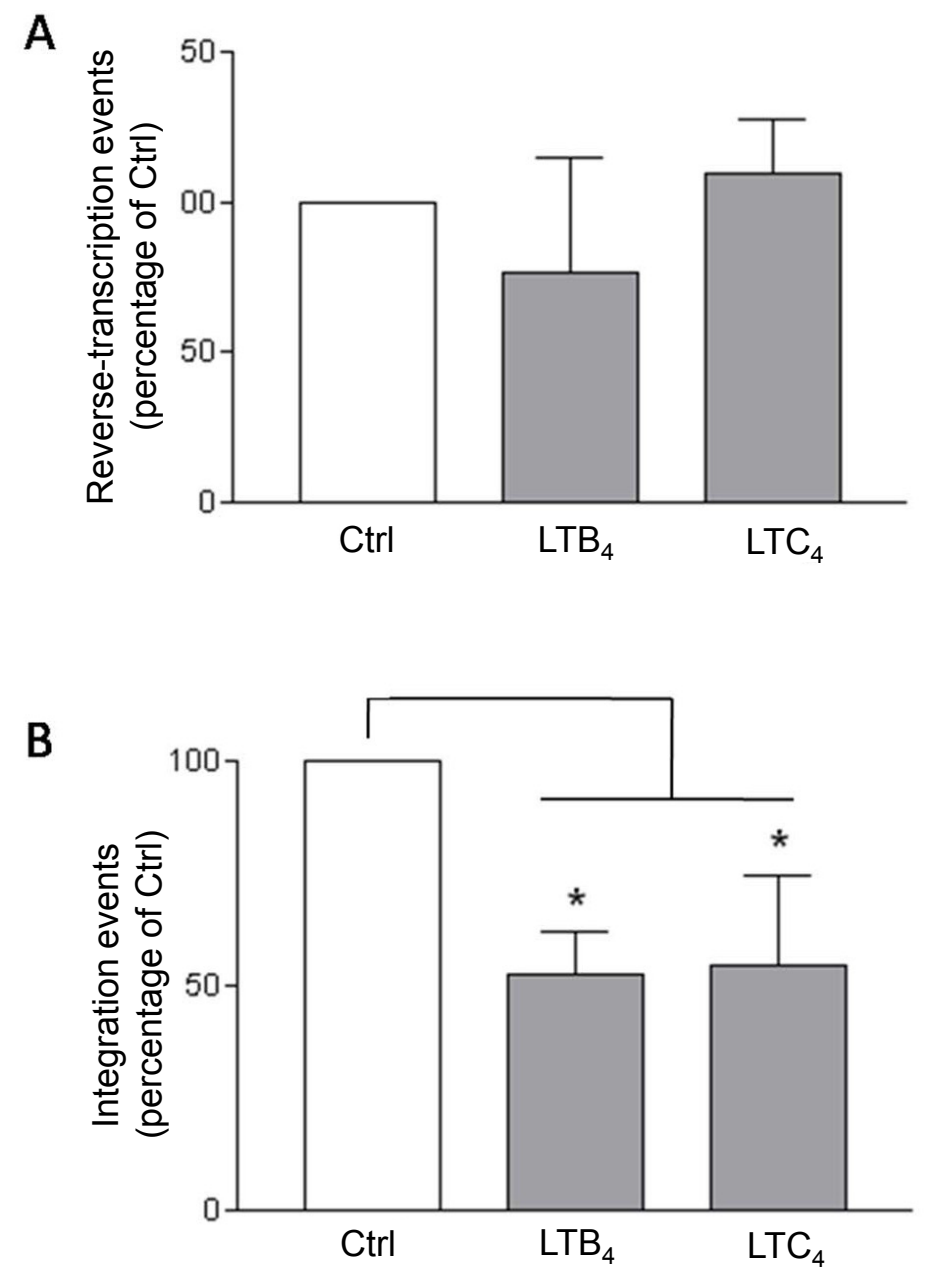
**Leukotrienes (LTs) have no impact on HIV-1 reverse transcripts, whereas they affect viral integration in monocyte-derived microglial-like cells (MDMis)**. Cells were first exposed to YU2 for 2 h. MDMis were then either left untreated (Ctrl) or treated with 10 ng/ml of LTB_4 _or LTC_4_. DNA was then extracted 8 h post infection for monitoring reverse transcribed products or 24 h post infection for assessing integration events. Quantification was achieved by real-time polymerase chain reaction (PCR). Amplified products were normalized using the housekeeping β-globin gene. To compensate for donor-to-donor variations, the results shown represent the means ± SD calculated from three independent experiments and are expressed as percentages of control (Ctrl) (*P < 0.05).

To gain insights on the nature of HIV-1 post-fusion events modulated by LTs, we then analyzed the reverse transcription and integration processes. Completed reverse transcripts and proviral DNA were quantified by real-time PCR in MDMis treated with LTs. Here, in order to distinguish the post-entry events from the consequences on the pH-independent viral entry at the plasma membrane, cells were treated with LTs at 2 h post infection. As shown in Figure 6, LTB_4 _and LTC_4 _had no significant impact on the amount of completed HIV-1 reverse transcription products, whereas both LTs induced a 50% reduction of integrated proviral DNA in MDMis.

### The effect of LTs on HIV-1 infection is reliant on PKC

**Figure 7:**
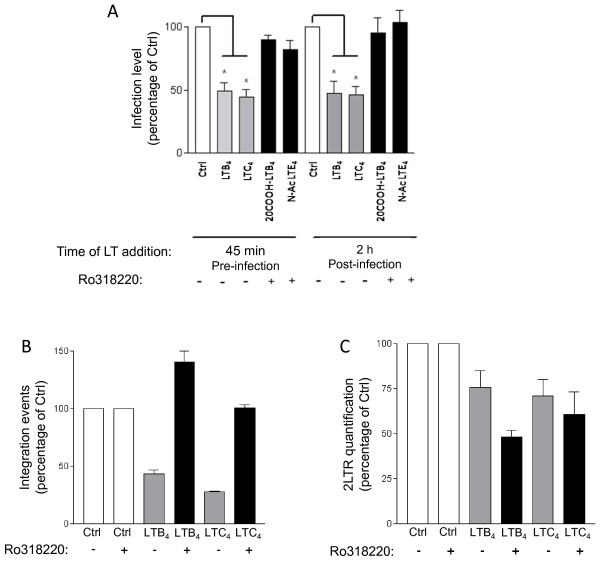
**Leukotrienes (LTs) reduce HIV-1 infection of monocyte-derived microglial-like cells (MDMis) in a protein kinase C (PKC)-dependent manner**. **(A) **MDMis were first either left untreated or treated with the large-spectrum PKC inhibitor Ro318220 (1 μM) for 30 minutes. LTs (10 ng/ml) were then added to cells either for 45 minutes before infection or after 2 h of incubation with JR-FL pseudotypes. Luciferase activity was measured 7 days post infection. **(B, C) **MDMis were first infected with vesicular stomatitis virus envelope glycoprotein G (VSV-G) pseudotypes for a 2 h period, cells were then treated with Ro318220 for 45 minutes, washed, and treated with LTs (100 ng/ml). Total DNA was extracted from cells in order to quantify HIV-1 integration events (panel B) and nuclear 2LTR circles **(C) **by real-time polymerase chain reaction (PCR). The results shown are expressed as percentages of control (that is, untreated cells) and calculated from five (A) or two (B, C) independent experiments in triplicate samples (*P < 0.05).

It is known that treatment of cells with LTs is often associated with PKC activation. Besides, Warrilow and coworkers have shown that some PKC modulatory compounds inhibit HIV-1 infection in PBMCs [62]. We therefore evaluated the involvement of PKC in the LT-mediated inhibitory effect on HIV-1 replication in MDMis. To do so, MDMis were treated, or not, with the large-spectrum PKC inhibitor Ro318220, prior to their exposure to luciferase-encoding HIV-1 particles. LTs were added on cells either 45 minutes before or 2 h after infection, and luciferase activity was then measured 7 days post infection. Results illustrated in Figure 7A demonstrate that the LT-induced modulation of HIV-1 infection in MDMis is abolished upon treatment with the PKC inhibitor, indicating that a PKC-mediated signal transduction pathway is involved in that phenomenon. Furthermore, the fact that the effect is observed whether LTs are added before or after infection indicates that PKC is involved in both early and post-fusion events of HIV-1 infection. To better define the involvement of PKC in the LT-modulated inhibitory effect on HIV-1 post-fusion events, we quantified both integration events and the formation of 2LTR circles in MDMis treated or not with the large spectrum PKC inhibitor Ro318220 prior to their exposure to LTs. It is important to note that the number of 2LTR circles serves as a quantitative measurement for nuclear import. For this set of experiments, VSV-G-pseudotyped viral particles were used in order to achieve a high infection efficiency that is necessary to detect the relatively rare 2LTR circle intermediates. As illustrated in Figure 7B, the reduction in intregrated proviral DNA in MDMis treated with the studied LTs is totally abolished following Ro318220 treatment. Moreover, a detectable reduction in the level of 2LTR circles was also observed in LT-treated MDMis, indicating a blockage at or before the nuclear import steps of the viral replication cycle (Figure 7C). However, Ro318220 failed to abolish this effect of LTs on 2LTR in MDMis, which points to another PKC-independent pathway that might also inhibit HIV-1 production in MDMis.

## Discussion

There is a paucity of data regarding the impact of potent eicosanoid lipid mediators on HIV-1 replication. Eicosanoids include four distinct families, that is, prostaglandins, prostacyclins, thromboxanes and LTs. Various studies have shown that prostaglandins can induce cellular resistance to HIV-1 [63-66]. As for LTs, only recently have we considered them as antimicrobial or anti-HIV-1 mediators [44,45,59,67-70]. In the CNS in particular, the interaction between LTs produced by immune cells and HIV-1 infection is poorly understood. We therefore focused our studies on the effect of LTs on HIV-1 infection in microglial cells. We chose to conduct our experiments in MDMis since this novel *in vitro *model mimics infiltrating microglia that are subjected to productive HIV-1 infection under *in vivo *situations.

To the best of our knowledge, other than one recent publication [71] no further studies on MDMi cells have been published following their initial characterization by Leone and colleagues in 2006 [55]. For this reason, we first validated our MDMis by verifying their morphology, IBA1 expression, substance P production and susceptibility to HIV-1 infection. We also verified the presence of LTB_4 _and LTC_4 _receptors on these cells. We were thus able to test this novel cellular model, which allowed us to overcome the difficulty of obtaining primary human microglial cells in sufficient quantities.

We determined that both LTB_4 _and LTC_4 _decrease productive HIV-1 infection of MDMis. More precisely, LTs seem to negatively modulate the earliest as well as post-fusion events. However, we were not able to observe any effects on late stages of infection (48 h post infection). The concentrations used in the current study seem higher than the LT concentrations found in the CSF in inflammatory conditions, which can vary between 116 and 348 pg/ml [32,37]. However, levels of LTs in CSF are probably an undervaluation of the amount produced in brain tissue. Indeed, because LTs can be secreted locally by glial cells, concentrations similar or higher to those used in our work are likely reached in the microenvironment surrounding brain microglial cells based on the notion that pg of LTs are measured per g of brain tissue.

We examined whether the HIV-1 coreceptor CCR5 was affected by the studied LTs. Since we did not observe any CCR5 modulation when MDMis were pre-exposed to LTs for 45 minutes, LTs likely do not cause CCR5 internalization by means of heterologous desensitization, which is a mechanism of rapid internalization of unstimulated G protein-coupled receptors that occurs despite the continued presence of a ligand. However, MDMis exposed to LTs for a longer period (24 h) showed a dose-dependent decrease in CCR5 surface expression. LT-treated MDMis might produce different β-chemokines (for example, macrophage inflammatory protein (MIP)-1α, MIP-1β and chemokine (C-C motif) ligand 5 (CCL5, also known as RANTES)), which may be responsible for the delayed CCR5 downmodulation by means of homologous desensitization partly responsible for the negative effect of LTs on the production of virion progeny. Further studies will be needed to pinpoint the exact mechanisms by which CCR5 is modulated by LTs. Besides, it is possible that MDMis exposed to LTs show attenuated levels of other surface molecules known to play a role in HIV-1 infection in the CNS, such as CCR3 or galactocerebroside (or galactosylceramide). Although controversial, it has been shown that LTB_4 _receptor could act as a coreceptor for HIV-1 in CD4-positive cells [72,73]. If true, competition for LT receptors might be one explanation for how LTs attenuate HIV-1 replication in MDMis.

The early stages of HIV-1 replication include all steps from binding at the cell surface to integration into the nucleus [74]. At the post-entry level, these early events consist of viral decapsidation, reverse transcription, nuclear import and integration. Interestingly, the strongest inhibition with respect to HIV-1 replication in MDMis was measured when target cells were exposed to LTs 2 h post infection, which is after virus entry. Thus, early post-entry events may also be targeted. We indeed detected lower amounts of integrated viral DNA in MDMis exposed to LTs 2 h following their infection, while the reverse-transcribed products remained unaffected. These data suggest that post-entry inhibition of HIV-1 infection by LTs in MDMis occurs between the reverse transcription and integration steps. Nuclear import of the HIV-1 genome is mediated by a central HIV-1 DNA flap that is formed at the end of reverse transcription [75]. This three-stranded DNA structure is necessary for the uncoating process that characterizes the maturation of reverse transcribed complexes into preintegration complexes [76]. Little is known about the regulation of such events by host cell factors. It is possible that signals transduced from LT receptors interfere with the formation of the DNA flap or the uncoating process. Further research will be needed to investigate these possibilities.

Interestingly, previous studies have shown that PKC modulatory compounds can affect the early events of HIV-1 infection in PBMCs [62]. Incubation of MDMis with a large spectrum PKC inhibitor abolished the effect of LTs on HIV-1 infection both at the early and post-fusion levels. PKC thus act as second messengers responsible for the LT-mediated reduction in HIV-1 infection. There are 12 PKC isozymes distributed either in the conventional (require calcium, diacylglycerol and phospholipids for activation), novel (require diacylglycerol for activation) or atypical (require neither calcium nor diacylglycerol for activation) subfamilies. Although it is not yet known which PKC isozymes are expressed in MDMis, LT stimulation could activate any of these PKC in this cellular model. Additional studies will be needed to identify which PKC isozymes and what downstream signals are implicated in the inhibition of HIV-1 in MDMis by LTs. To determine more precisely which post-fusion step is inhibited by LTs in MDMis via PKC, we measured integrated viral DNA and nuclear 2LTR circles. We found that integrated proviral DNA is diminished by LTs in a PKC-dependent manner while 2LTR levels are decreased independently of the PKC signaling pathway in MDMis. Consequently, it can be proposed that LTs block the integration process after formation of the 2LTR circles in the nucleus by a mechanism that is partly mediated by PKC, but a reduction of the nuclear import via a PKC-independent pathway cannot be excluded. Nuclear import of the HIV-1 preintegration complex depends on members of the cell nuclear transport machinery such as karyopherin β transportin-3 (TNPO3) and nucleoporin 153 (NUP153) [77-79]. Interestingly, this nuclear transport machinery is inhibited by phosphorylation via mitogen-activated protein kinase kinase (MEK) and phosphatidylinositol 3-kinase signaling [80]. It is possible that such signal transduction pathways are induced by LT treatment, causing nucleoporin phosphorylation and inhibition of nuclear import. Additional studies are needed to solve this issue.

## Conclusions

In summary, the goal of this study was to analyze the possible involvement of LTs in the control of HIV-1 infection in microglia. The results of this study indicate that LTB_4 _and LTC_4 _inhibit some early events of the HIV-1 replication cycle in MDMis and that this effect relies on PKC. Now, if confirmed *in vivo*, what physiological relevance would be presented by the observed attenuation of HIV-1 infection by LTs? In the post-HAART era, individuals still show mild symptoms of neuroinflammation associated to HIV-1 infection. LTs, among other proinflammatory molecules, are often found in the CSF of patients who are infected with HIV-1. Because of the high selectivity and low permeability of the BBB, the CNS is under weak immunological surveillance. However, viral load in the CNS is rarely much higher than in peripheral blood. Neuroinflammation, accompanied by production of LTs, may play a protective role against viral attack such as HIV-1. Since microglia are largely responsible for productive HIV-1 infection in the CNS, the data presented here provide an insight into how LTs may be one factor that help control viral load in the CNS compartment. Although some previous studies reported no adverse side effects associated with intravenous injections of LTB_4 _to humans or monkeys [44,45], additional research will be needed to determine if LTs can be used as a treatment or prophylaxis for HIV-1 infection. Thus, experimental studies using *in vivo *models will be needed to clarify the exact role of these proinflammatory lipid mediators in the modulation of viral load in the CNS.

## Competing interests

The authors declare that they have no competing interests.

## Authors' contributions

JB carried out most experiments described in the manuscript and drafted the manuscript. CB participated in the design and coordination of the study and helped to draft the manuscript. DB developed the cell culture system described in this work. MJT participated in the design and coordination of the study and helped to draft the manuscript. All authors read and approved the final manuscript.
